# Detection of *TP53* Clonal Variants in Papanicolaou Test Samples Collected up to 6 Years Prior to High-Grade Serous Epithelial Ovarian Cancer Diagnosis

**DOI:** 10.1001/jamanetworkopen.2020.7566

**Published:** 2020-07-01

**Authors:** Lara Paracchini, Chiara Pesenti, Martina Delle Marchette, Luca Beltrame, Tommaso Bianchi, Tommaso Grassi, Alessandro Buda, Fabio Landoni, Lorenzo Ceppi, Cristina Bosetti, Mariachiara Paderno, Marco Adorni, Debora Vicini, Patrizia Perego, Biagio Eugenio Leone, Maurizio D’Incalci, Sergio Marchini, Robert Fruscio

**Affiliations:** 1 Department of Oncology, Istituto di Ricerche Farmacologiche Mario Negri IRCCS, Milan, Italy; 2 Department of Obstetrics and Gynecology, Università degli Studi Milano-Bicocca, San Gerardo Hospital, Monza, Italy; 3 Department of Obstetrics and Gynecology, Azienda Socio Sanitaria Territoriale -Monza, Desio Hospital, Desio, Italy; 4 Department of Pathology, Università degli Studi Milano-Bicocca, San Gerardo Hospital, Monza, Italy

## Abstract

**Importance:**

The low 5-year survival rate of women with high-grade serous epithelial ovarian cancer (HGS-EOC) is related to its late diagnosis; thus, improvement in diagnosis constitutes a crucial step to increase the curability of this disease.

**Objective:**

To determine whether the presence of the clonal pathogenic *TP53* variant detected in matched primary tumor biopsies can be identified in DNA purified from Papanicolaou test samples collected from women with HGS-EOC years before the diagnosis.

**Design, Setting, and Participants:**

This cohort study was conducted among a single-center cohort of women with histologically confirmed diagnosis of HGS-EOC recruited at San Gerardo Hospital, Monza, Italy, from October 15, 2015, to January 4, 2019. Serial dilutions of DNA derived from tumor samples and DNA extracted from healthy women’s Papanicolaou test samples were analyzed to define the sensitivity and specificity of droplet digital polymerase chain reaction assays designed to detect the *TP53* variants identified in tumors. All available brush-based Papanicolaou test slides performed up to 6 years before diagnosis were investigated at the Mario Negri Institute, Milano, Italy. Data were analyzed from October 2018 to December 2019.

**Main Outcomes and Measures:**

The presence of tumor pathogenic *TP53* variants was assessed by the droplet digital polymerase chain reaction approach in DNA purified from Papanicolaou test samples obtained from the same patients before diagnosis during cervical cancer screenings.

**Results:**

Among 17 included patients (median [interquartile range] age at diagnosis, 60 [53-69] years), Papanicolaou tests withdrawn before diagnosis presented tumor-matched *TP53* variants in 11 patients (64%). In 2 patients for whom longitudinal Papanicolaou tests were available, including 1 patient with Papanicolaou tests from 25 and 49 months before diagnosis and 1 patient with Papanicolaou tests from 27 and 68 months before diagnosis, the *TP53* clonal variant was detected at all time points.

**Conclusions and Relevance:**

These findings suggest that noninvasive early molecular diagnosis of HGS-EOC is potentially achievable through detection of *TP53* clonal variants in the DNA purified from Papanicolaou tests performed during cervical cancer screening.

## Key Points

**Question:**

Can clonal *TP53* variants be detected in Papanicolaou tests performed several years before high-grade serous epithelial ovarian cancer (HGS-EOC) diagnosis?

**Findings:**

This cohort study including 17 patients with HGS-EOC found that in 11 patients, tumor-specific *TP53* variants were detected in Papanicolaou tests performed up to 6 years before the diagnosis of HGS-EOC.

**Meaning:**

These findings suggest that very early diagnosis of HGS-EOC is potentially achievable and that further developments in highly sensitive molecular approaches could improve early diagnosis of HGS-EOC.

## Teaser

This cohort study assesses whether *TP53* clonal variants can be detected in Papanicolaou test samples from women later diagnosed with high-grade serous epithelial ovarian cancer (HGS-EOC).

## Introduction

High-grade serous epithelial ovarian cancer (HGS-EOC) is characterized by a clonal pathogenic variant in the *TP53* (OMIM 191170) gene, which represents one of the early events in the etiopathogenetic process.^1,2^ It has been demonstrated that the same *TP53* clonal variant detected in the primary tumor site can also be detected in precancerous lesions in the Fallopian tube, known as serous tubal intraepithelial carcinomas.^3^ Recently, early serous proliferations in the Fallopian tube were found to share the same *TP53* variant identified in concurrent metastatic HGS-EOCs, even in the absence of a detectable serous tubal intraepithelial carcinomas.^4^ A mathematical model based on a lesion-specific proliferation rate suggests that serous tubal intraepithelial carcinomas progression to carcinoma takes approximately 6 years. To our knowledge, experimental evidence in support of this theoretical prediction is lacking,^3^ which hampers the potential development of a test for the early diagnosis of ovarian cancer.^3^

Clonal pathogenic variants in the *TP53* gene are suitable candidates to identify early steps in the neoplastic transformation toward HGS-EOC at the molecular level. Recently, several studies^5,6,7^ have shown the feasibility of detecting somatic variants in DNA from endometrial and ovarian cancers retrieved from various types of vaginal samples collected at the time of diagnosis. The aim of this study was to explore the possibility of exploiting the Papanicolaou test conducted for cervical cancer screening years before diagnosis as a source of material to detect clonal variants in the *TP53* gene as a basis to develop assays for the early diagnosis of HGS-EOC.

## Methods

This study was approved by the ethics committee of San Gerardo Hospital, Monza, Italy. All participants provided signed informed consent, and participants did not receive any financial compensation. This study followed the Strengthening the Reporting of Observational Studies in Epidemiology (STROBE) reporting guideline for cohort studies.

Women with histologically confirmed stage II through IV HGS-EOC were selected from a collection of patients with HGS-EOC who underwent primary surgical treatment at the San Gerardo Hospital from October 15, 2015, to January 4, 2019. As described in the Figure, women were selected on the basis of the availability of primary tumor formalin-fixed paraffin-embedded slides and matched brush-based Papanicolaou test slides routinely withdrawn during cervical cancer screenings performed at different time points before diagnosis (up to 6 years) and cytologically negative for dysplasia or any other malignant neoplasms.

**Figure.**
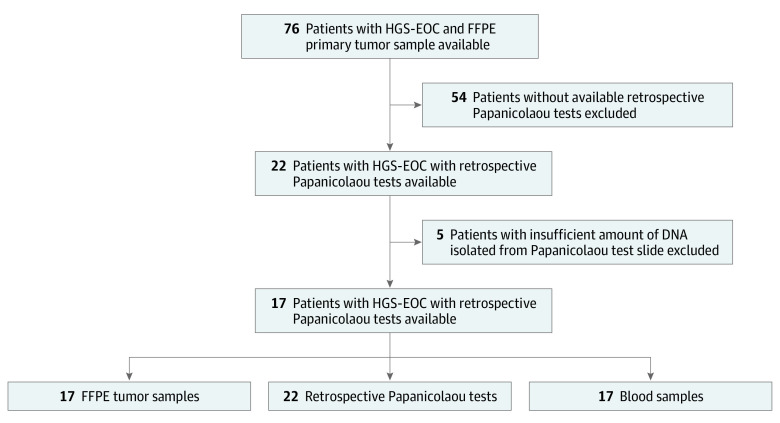
Study Flow Diagram For each locus-specific *TP53* variant assay, droplet digital polymerase chain reaction experiments were performed with both positive and negative controls. Assay sensitivity was assessed on DNA purified from healthy donors. FFPE indicates formalin-fixed paraffin-embedded and HGS-EOC, high-grade serous epithelial ovarian cancer.

To identify the somatic clonal pathogenic *TP53* variants in primary tumor biopsies, DNA was purified from formalin-fixed paraffin-embedded samples (eAppendix 1 and eFigure in the Supplement) and was analyzed by next generation sequencing (NGS) technology following manufacturer’s instructions. Briefly, NGS libraries were prepared starting from 100 ng of tumor DNA, using a capture-based custom panel of probes that covers the exons of the *TP53* gene (SeqCap EZ Target Enrichment System, Roche). After the barcoding procedure, samples were pooled and sequenced on the NextSeq 500 Platform (Illumina) with a median (interquartile range) coverage of 17386X (8157X-27355X) (eAppendix 2 and eTable 1 in the Supplement). Subsequently, bioinformatic analyses were performed to identify the clonal pathogenic variant in each tumor sample. Annotation and interpretation of the *TP53* variants were performed using dbSNP,^8^ COSMIC,^9^ ClinVar,^10^ and IARC TP53^11^ databases. The identified somatic clonal *TP53* variants were classified as pathogenic, likely pathogenic, or variant of unknown significance and mostly mapped in *TP53* variant hot spots^12^ (eTable 1 in the Supplement). Detailed description of the experimental protocols and bioinformatic pipelines are reported in the eAppendix 2 in the Supplement.

The clonal pathogenic *TP53* variants identified by the NGS approach in the tumor biopsy were then orthogonally validated in the patient’s tumor biopsy and were investigated in matched Papanicolaou tests by droplet digital polymerase chain reaction (ddPCR) experiments as detailed in eAppendix 3 in the Supplement. Briefly, for each *TP53* somatic variant, a locus-specific ddPCR variant assay was designed according to the online BioRad tool (eTable 3 in the Supplement), and the limit of detection of each assay was experimentally evaluated by serial dilution experiments. Experiments were performed on a QX200 ddPCR system (BioRad) with the support of QX200 AutoDG Droplet Digital PCR System (BioRad). To reduce interexperiment and intraexperiment droplet generation variability, ddPCR analysis was run on DNA extracted from patients’ Papanicolaou test slides; matched tumor DNA, as positive control; DNA extracted from matched blood sample, as negative control; and DNA purified from healthy women’s Papanicolaou tests to assess the specificity of the assays. To this end, Papanicolaou tests from healthy women without personal history or genetic predisposition for hereditary cancers were included in the study. Selected women underwent surgery for benign conditions and Papanicolaou tests were collected at the time of surgery.

### Statistical Analysis

For selected continuous variables, mean and SD or median and interquartile range values and their corresponding measures of dispersion were provided. Statistical analyses were carried out with GraphPad Prism, version 8.4.2 (GraphPad).

## Results

The Figure depicts the selection of HGS-EOC patients enrolled in the study, while Table 1 details the main clinical and pathological features for each patient, as well as the number and the temporal windows before the diagnosis at which each Papanicolaou test was withdrawn. Briefly, 17 patients with histologically confirmed diagnosis of HGS-EOC (International Federation of Gynecology and Obstetrics stage II-IV) who underwent debulking surgical treatment at the Department of Obstetrics and Gynecology of San Gerardo Hospital were retrospectively selected for this study. The median (interquartile range) age at diagnosis was 60 (53-69) years (Table 1). The prevalence of *BRCA1* (OMIM 113705) and *BRCA2* (OMIM 600185) germline variants was 58.8% (10 patients): 8 patients carried a *BRCA1* variant and 2 patients carried a *BRCA2* variant (Table 1).

**Table 1.** Description of Clinicopathological Characteristics of Women With High-Grade Serous Epithelial Ovarian Cancer

Patient ID^a^	FIGO stage	Age at diagnosis, y	Date of primary surgical treatment, y	Germinal status of *BRCA*	Time of Papanicolaou test, mo before surgical treatment^b^			
					T1	T2	T3	T4
21561	IIIC	60	2016	WT	0.2	NA	25	49
21585	IIA	42	2017	BRCA1	NA	11.3	NA	NA
21567	IIIC	48	2016	BRCA1	3	NA	NA	NA
21587	IVA	65	2017	WT	2	NA	NA	NA
21586	IIIB	72	2017	BRCA1	NA	19.3	NA	NA
21569	IIIC	81	2016	WT	5.2	NA	NA	NA
21624	IIIC	69	2017	WT	NA	NA	37.5	65.3
21570	IIIC	70	2016	WT	0.3	NA	NA	NA
21627	IIIC	54	2017	BRCA2	0.7	NA	NA	NA
21640	IVA	53	2018	WT	NA	8	NA	NA
21507	IIIC	57	2015	BRCA1	NA	9.2	NA	NA
21635	IIIC	60	2018	BRCA2	1.3	NA	NA	NA
21549	IIIC	70	2016	BRCA1	NA	NA	31.2	65.3
21521	IIIB	61	2016	BRCA1	NA	NA	26.7	67.3
21654	IIIC	59	2018	BRCA1	4.7	NA	NA	NA
21665	IIIC	47	2019	BRCA1	NA	NA	37.6	NA
21683	IVC	53	2018	WT	NA	18.5	NA	NA

Abbreviations: BRCA1, presence of a germline pathogenic variant in *BRCA1*; BRCA2, presence of a germline pathogenic variant in *BRCA2*; FIGO, International Federation of Gynecology and Obstetrics; NA, not available; T, time point; WT, wild-type.

^a^ For anonymity, individual patients are identified by number.

^b^ Time points are arbitrary temporal windows used to describe the time before diagnosis at which each Papanicolaou tests was available. T1 indicates 0 to 6 months; T2, 7 to 24 months; T3, 25 to 48 months; T4, 49 months or longer.

A total of 22 Papanicolaou test samples performed up to approximately 6 years before HGS-EOC diagnosis were collected. For 4 patients (23.5%) more than 1 Papanicolaou test sample was available for analysis; among these, 3 patients provided 2 Papanicolaou test samples, while 3 Papanicolaou test samples were available for 1 patient only. The median (interquartile range) interval from Papanicolaou test collection to HGS-EOC diagnosis was 14.9 (3.4-35.9) months.

Analysis of the somatic single nucleotide variants in the exonic regions of the *TP53* gene by NGS technology allowed identification of the clonal *TP53* variant in primary tumor samples of each patient. The complete list of identified variants with their allelic frequency is shown in Table 2, and further details are presented in eTable 1 in the Supplement.

**Table 2.** List of *TP53* Clonal Variants Identified in Primary Tumor Biopsies

Patient ID^a^	*TP53* variant	Significance	In variant hot spot	NGS variant fraction, %	ddPCR relative abundance, %
21561	c.818G>A p.R273H	P	Yes	65.01	69.91
21585	c.817C>T p.R273C	VUS	Yes	40.94	44.63
21567	c.281C>A p.S94*	NR	No	71.98	69.51
21587	c.469G>T p.V157F	LP	Yes	15.12	15.55
21586	c.818G>A p.R273H	P	Yes	79.73	82.97
21569	c.574C>T p.Q192*	P	Yes	62.36	64.10
21624	c.820G>T p.V274F	LP	Yes	86.72	93.37
21570	c.844C>T p.R282W	P	Yes	89.19	90.94
21627	c.425_427del p.P142_V143del_insL	NR	Nr	76.04	80.92
21640	c.993 + 2T>G	NR	No	70.68	70.67
21507	c.1025G>C p.R342P	P	Yes	91.23	93.75
21635	c.844C>T p.R282W	P	Yes	66.24	65.98
21549	c.393_395del p.N131del	VUS	Yes	54.06	67.01
21521	c. 722 C>G p.S241C	LP	Yes	61.15	69.22
21654	c.586 C>T p.R196*	P	Yes	49.35	34.03
21665	c.393_395del p.N131del	VUS	Yes	45.34	55.05
21683	c.602 *t* > A p.L201*	NR	No	33.42	35.22

Abbreviations: ddPCR, droplet digital polymerase chain reaction; LP, likely pathogenic; NGS, next generation sequencing; NR, not reported; P, pathogenic; VUS, variant of unknown significance.

^a^ For anonymity, individual patients are identified by number.

For each patient, we investigated the presence of *TP53* clonal variants by ddPCR technique in DNA purified from matched Papanicolaou tests samples obtained months or years before diagnosis. Since we aimed to detect variants with very low allelic frequency, the relative abundance (RA) percentage of the analyzed *TP53* variant was calculated according to 2 stringent parameters: only droplets containing DNA harboring *TP53* variants marked by the fluorescein fluorophore were considered positive and each sample had to show fluorescein-marked droplets in all independent replicates, otherwise results were considered irreproducible artifacts (eAppendix 3 in the Supplement). Based on results from serial dilution sensitivity tests (eTable 2 in the Supplement), all ddPCR assays but 1 (ie, *TP53* c.469G>T) were able to detect approximately 0.1% to 0.05% of tumor content with variable RA, depending on the starting tumor variated allele frequency. The assay for *TP53* c.817C>T allowed detection down to 0.01% (eTable 2 in the Supplement). Eleven Papanicolaou tests from healthy women were analyzed as negative controls using the equivalent DNA source, to assess assay specificity. None of these samples was positive for *TP53* variants identified in our tumor cohort (eTable 3 in the Supplement).

For 11 of 17 patients (64%), the *TP53* clonal variant was also detectable in all matched Papanicolaou tests collected within 6 months before diagnosis (Time point [T] 1) or earlier (T2, T3, and T4) (Table 3). The RA percentages were always higher than the limit of detection established by the sensitivity tests. For 3 patients, 2 or more Papanicolaou tests conducted at different times before diagnoses were available (Table 1). For 1 patient, the *TP53* clonal variant was identified in all 3 Papanicolaou tests, performed 9 days (T1, RA = 0.24%), 25 months (T3, RA = 0.21%), and 49 months (T4, RA = 0.26%) prior to diagnosis (Table 3). Among 2 patients with 2 samples each, the *TP53* variant was confirmed 27 months (T3, RA = 0.05%) and 68 months (T4, RA = 0.07%) before diagnosis for 1 patient and in only 1 of the available Papanicolaou tests for the other patient (T3, RA = 0.04%) (Table 3).

**Table 3.** Droplet Digital Polymerase Chain Reaction Results on Patients’ Papanicolaou Tests

Patient ID^a^	*TP53* variant	Papanicolaou tests, % relative abundance^b^			
		T1	T2	T3	T4
21561	c.818G>A p.R273H	0.24	NA	0.21	0.26
21585	c.817C>T p.R273C	NA	0.21	NA	NA
21567	c.281C>A p.S94*	0.07	NA	NA	NA
21587	c.469G>T p.V157F	ND	NA	NA	NA
21586	c.818G>A p.R273H	NA	0.15	NA	NA
21569	c.574C>T p.Q192*	1.18	NA	NA	NA
21624	c.820G>T p.V274F	NA	NA	0.04	ND
21570	c.844C>T p.R282W	2.62	NA	NA	NA
21627	c.425_427del p.P142_V143del_insL	2.4	NA	NA	NA
21640	c.993 + 2T>G	NA	ND	NA	NA
21507	c.1025G>C p.R342P	NA	9.2	NA	NA
21635	c.844C>T p.R282W	ND	NA	NA	NA
21549	c.393_395del p.N131del	NA	NA	ND	ND
21521	c. 722 C>G p.S241C	NA	NA	0.05	0.07
21654	c.586 C>T p.R196*	0.09	NA	NA	NA
21665	c.393_395del p.N131del	NA	NA	ND	NA
21683	c.602 *t* > A p.L201*	NA	0.06	NA	NA

Abbreviations: NA, not available; ND, not detected.

^a^ For anonymity, individual patients are identified by number.

^b^ Time points are arbitrary temporal windows used to describe the time before diagnosis at which each Papanicolaou tests was available. T1 indicates 0 to 6 months; T2, 7 to 24 months; T3, 25 to 48 months; T4, 49 months or longer.

## Discussion

This cohort study found that *TP53* clonal somatic variants found at the ovarian cancer site were detectable in the same patients’ archival Papanicolaou tests performed up to 6 years before tumor diagnosis. Remarkably, for 2 of 3 patients for whom 2 or more archival Papanicolaou tests were available, the same clonal *TP53* variant was confirmed in all samples. Moreover, although most of the *TP53* variants were located in variant hot spots, they were not found in healthy women’s samples, corroborating the etiopathogenetic role of selected *TP53* variants in patients with HGS-EOC. To our knowledge, this is the first experimental evidence that supports the mathematical model according to which HGS-EOC takes at least approximately 6 years to develop.^3^ Considering the anatomical continuity between tubal lumen and cervical canal, it is plausible that cytological material could be a useful biological material to detect biomarkers associated with HGS-EOC many years before diagnosis, although a study focused on HGS-EOC etiopathogenesis is necessary to assess the viability of this model.

The detectability of *TP53* clonal variants in Papanicolaou test samples taken at the time of ovarian cancer diagnosis has been shown previously.^5,6,7^ A 2019 study by Arildsen et al^13^ reported that *TP53* variants detected by ddPCR were found in 7 diagnostic liquid-based Papanicolaou tests from 15 HGS-EOCs. Arildsen et al^13^ detected *TP53* clonal variant in 1 patient’s Papanicolaou test performed 20 months before diagnosis. The discrepancy between these findings and our results is possibly due to technical issues, such as the likelihood that the DNA stability in liquid-based archival Papanicolaou tests was lower than that of our samples, which were brush-based and stored dry. These data suggest that it is possible to use the Papanicolaou test for early diagnosis of HGS-EOC.

### Strengths and Limitations

This study has 2 main strengths. To our knowledge, this is the first investigation in which Papanicolaou tests from healthy women were used as controls to investigate the presence of *TP53* variants in the same biological material analyzed in patients with HGS-EOC. The absence in the control samples of the *TP53* variants identified in tumor DNA supports our hypothesis that the presence of these *TP53* variants in patients’ archival Papanicolaou test samples represents an early sign of disease. Additionally, despite stringent application of ddPCR analysis criteria to limit the inclusion of potentially false-positive results, we obtained a detection rate close to 64%.

This study also has limitations. The main limitations of the study are that it is a proof-of-principle study, conducted in a small cohort of patients with Papanicolaou tests collected at different time points for each patient and with a low degree of overlap among patients. Indeed, the retrieval of the material for this study was difficult, as Papanicolaou test screening was based on women’s voluntary participation, and Papanicolaou tests were usually conducted in different laboratories during the course of the study. Secondly, in 5 of 17 patients, Papanicolaou test results were negative for the *TP53* variants. For 1 patient with 2 Papanicolaou tests available, the *TP53* variant was detected in only 1 Papanicolaou test. Thus, the detection rate was calculated only on 11 patients with all Papanicolaou tests positive for *TP53* variants. Since the archival Papanicolaou test material was not originally meant to be used for DNA analysis, sampling procedures and storage conditions could have interfered with DNA quality. Indeed, the quality of DNA from the healthy controls, processed immediately after administration, was superior (eFigure in the Supplement). Therefore, it is conceivable that the negative findings for *TP53* variants are, at least in part, due to inadequate sample collection and storage. Another limitation worth noting is that we evaluated only tumor-matched *TP53* clonal variants. Somatic evolution in nonneoplastic tissues implies that, owing to aging or other physiological processes, multiple somatic variants are normally present ubiquitously at the mosaic level.^14,15^ Therefore, prospective clinical studies conceived and designed on large populations of women are required to validate our findings.

## Conclusions

This cohort study found specific variants in multiple Papanicolaou tests from the same patients conducted up to 6 years before the diagnosis of HGS-EOC. The identified variants were mostly located in variant hot spots. The development of a clinically and analytically accurate diagnostic test will require a large, longitudinal prospective study to be conducted with appropriate numbers of patients and healthy controls using standardized sampling procedures and highly sensitive NGS-based approaches to monitor the entire *TP53* gene. Women harboring *BRCA1* or *BRCA2* germinal variants could be suitable candidates to be recruited into such a study, given their high risk of HGS-EOC and their intensive monitoring. Furthermore, only such ad hoc studies will ultimately allow a robust evaluation as to whether this early diagnostic approach might translate into survival benefits. Our results hint at a promising prospect to significantly improve the future diagnosis of HGS-EOC, thus increasing its potential curability.

## Notes

Supplement. **eAppendix 1.** DNA Extraction **eFigure.** Distribution of DNA Concentration From Tumor FFPE Samples, Blood Samples, and Papanicolaou Tests From Patients and Healthy Women **eAppendix 2.** Tumor Next Generation Sequencing **eTable 1.** Summary Description of *TP53* Clonal Somatic Variants Identified by Next Generation Sequencing **eAppendix 3.** Droplet Digital Polymerase Chain Reaction Experiments **eTable 2.** Serial Dilutions Experiments **eTable 3.** Assay Specificity **eReferences**
