# Supplementary material for: Detection of TP53 Clonal Variants in Papanicolaou Test Samples Collected up to 6 Years Prior to High-Grade Serous Epithelial Ovarian Cancer Diagnosis
Source: JAMA Netw Open. 2020 Jul 1;3(7):e207566. doi: 10.1001/jamanetworkopen.2020.7566 (PMC7330718; doi:10.1001/jamanetworkopen.2020.7566)
Supplement: Supplement. — eAppendix 1. DNA Extraction eFigure. Distribution of DNA Concentration From Tumor FFPE Samples, Blood Samples, and Papanicolaou Tests From Patients and Healthy Women eAppendix 2. Tumor Next Generation Sequencing eTable 1. Summary Description of TP53 Clonal Somatic Variants Identified by Next Generation Sequencing eAppendix 3. Droplet Digital Polymerase Chain Reaction Experiments eTable 2. Serial Dilutions Experiments eTable 3. Assay Specificity eReferences [file jamanetwopen-3-e207566-s001.pdf]

Supplementary Online Content

Paracchini L, Pesenti C, Delle Marchette M, et al. Detection of *TP53* clonal variants in Papanicolaou test samples collected up to 6 years prior to high-grade serous epithelial ovarian cancer diagnosis. *JAMA Netw Open*. 2020;3(7):e207566. doi:10.1001/jamanetworkopen.2020.7566

eAppendix 1. DNA Extraction
eFigure. Distribution of DNA Concentration From Tumor FFPE Samples, Blood Samples, and Papanicolaou Tests From Patients and Healthy Women
eAppendix 2. Tumor Next Generation Sequencing
eTable 1. Summary Description of *TP53* Clonal Somatic Variants Identified by Next Generation Sequencing
eAppendix 3. Droplet Digital Polymerase Chain Reaction Experiments
eTable 2. Serial Dilutions Experiments
eTable 3. Assay Specificity
eReferences

This supplementary material has been provided by the authors to give readers additional information about their work.

© 2020 Paracchini L et al. *JAMA Network Open.*

eAppendix 1. DNA Extraction

For each high grade serous epithelial ovarian cancer **(**HGS-EOC) patient, Formalin Fixed Paraffin Embedded (FFPE) sample was selected by pathologists and tumor DNA purified using Maxwell RSC DNA FFPE kit (Promega, Milan Italy) following the protocol instruction. DNA was extracted from brush-based Pap Tests slides stained with hematoxylin and eosin, both from patients and healthy women, using the QIAamp DNA FFPE Tissue kit (Qiagen, Milan Italy) without deparaffinization step. In order to preserve the original morphological findings of the smears, the slides were previously digitally scanned by a slide scanner (Aperio Scanscope) and the virtual slides are currently kept in the digital archive of the Department of Pathology of San Gerardo Hospital (Monza, Italy). DNA was isolated from blood samples by QIAamp DNA Mini kit (Qiagen) following manufacturer's instruction. DNA concentrations were assessed by Qubit High Sensitivity DNA assay (Thermo Fisher, Milano Italy) and their values are distributed as reported as in the **eFigure**.

eFigure. Distribution of DNA Concentration From Tumor FFPE Samples, Blood Samples, and Papanicolaou Tests From Patients and Healthy Women

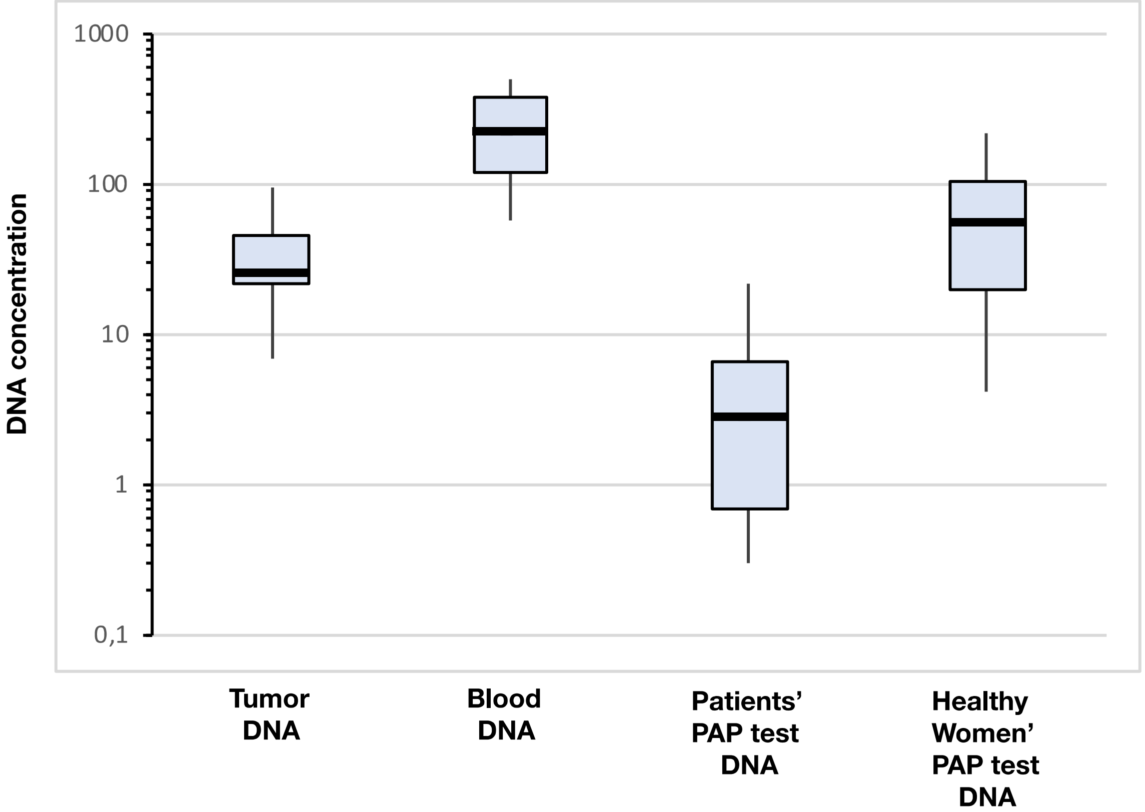

Data are plotted on log10 scale. The black lines indicate the median of the distribution; the margins in the boxes the 25th and the 75th percentile; the topmost and bottommost limits of the lines indicate the 5th and the 95th percentiles.

© 2020 Paracchini L et al. *JAMA Network Open.*

eAppendix 2. Tumor Next Generation Sequencing

For each tumor DNA sample, 100 ng of DNA were enzymatically fragmented and Whole Genome Libraries were made using the KAPA Hyper Plus kit (Roche) and following the SeqCap EZ HyperCap Workflow (Roche). Single indexes from KAPA Single-Indexed Adapter Kit (Roche) were assigned to each tumor DNA. All tumor DNA whole genome libraries were equimolarly pooled to a single pool with a combined mass of 1µg and hybridized for at least 18 hours with a custom probes panel that covers the exon regions of seven genes (*TP53, BRCA1, BRCA2, PTEN, CDK12, RAD51D, NF1*). The captured pool was then washed and amplified with seven cycles of PCR, following the manufacturer's instructions, then loaded on a NextSeq 500 sequencer (Illumina) and sequenced with an Illumina Sequencing Mid Output kit (300 cycles; 2x150bp).

The data were then demultiplexed with the bcl2fastq software (Illumina) and prepared for analysis. Raw reads were aligned to the reference genome (hg38) with the Burrows-Wheeler Aligner[1] (BWA) and then somatic variants were called with MuTect 2[2] and VarDict[3], merging the data in a final "ensemble" call set with results from both samples. A pool of unrelated, process-matched controls was used as normal to remove likely germline variants. Additional annotation of the variants was performed with the Variant Effect Predictor[4] (VEP; version 89) and an additional priority filter was used by checking different database entries on the identified variants: dbSNP (https://www.ncbi.nlm.nih.gov/snp/), COSMIC (https://cancer.sanger.ac.uk/cosmic), ClinVar (https://www.ncbi.nlm.nih.gov/clinvar/) to remove more germline variants. The resulting call set was loaded into a GEMINI[5] compatible database for downstream processing. Synonymous variants, and variants with less than 10% allelic fraction (AF) or with a variant depth of less than 200X were discarded. Raw data are available in the European Genone-Phenome Archive (EGA) under controlled access (EGAS00001004361).

The interpretation of each variant in the final call set was completed taking into account the frequency in the general population (gnomAD; https://gnomad.broadinstitute.org), its presence in database such as COSMIC and dbSNP and from data retrieved from the IARC TP53 database[6] (http://p53.iarc.fr).

In each sample, a *TP53* clonal somatic pathogenic variant was identified. The complete description of each variant is reported in **eTable 1**.

© 2020 Paracchini L et al. *JAMA Network Open.*

eTable 1. Summary Description of TP53 Clonal Somatic Variants Identified by Next Generation Sequencing

For each tumor biopsy is reported the identified *TP53* somatic variant, with its main information. Start and End refer to the genomic position of the selected variant according to the Human genome (assembly GRCh38/hg38). Ref, reference sequence; Alt, variant sequence; Type, the type of base change, SNV, single nucleotide variant, indel, small insertion or deletion. Impact, refers to the effect of mutation on the protein., M, missense variant; S, stop gained variant; ID, in frame deletion variant; SD, splice donor variant; dbSNP ID refers to the record of the variant reported in dbSNP database, COSMIC ID refers to the record of the variant reported in COSMIC database, Depth, refers to the coverage obtained after NGS run NA, not available; NR, not reported; Alt %, mutated fraction percentage.

Tumor	Start	End	Ref	Alt	Type	Impact	dbSNP ID	COSMIC ID	Depth	Alt %
21561_T	7673801	7673802	C	T	SNV	M	rs28934576	COSM99729	10868	65.01
21585_T	7673802	7673803	G	A	SNV	M	rs121913343	COSM99933	16866	40.94
21567_T	7676087	7676088	G	T	SNV	S	NR	COSM3733627	3815	71.98
21587_T	7675142	7675143	C	A	SNV	M	rs121912654	COSM1679513	43206	15.12
21586_T	7673801	7673802	C	T	SNV	M	rs28934576	COSM99729	3868	79.73
21569_T	7674956	7674957	G	A	SNV	S	rs866380588	COSM117946	21486	62.36
21624_T	7673799	7673800	C	A	SNV	M	rs1057520005	COSM1679490	28249	86.72
21570_T	7673775	7673776	G	A	SNV	M	rs28934574	COSM10704	15576	89.19
21627_T	7675183	7675187	ACAG	A	indel	ID	NR	NR	35557	76.04
21640_T	7673532	7673533	A	C	SNV	SD	NR	COSM4782217	2548	70.68
21507_T	7670683	7670684	C	G	SNV	M	rs375338359	COSM437468	17386	91.23
21635_T	7673775	7673776	G	A	SNV	M	rs28934574	COSM10704	18012	66.24
21549_T	7675215	7675219	CTTG	C	indel	ID	rs879254214	COSM4735425	27355	54.06
21521_T	7674240	7674241	G	C	SNV	M	rs28934573	COSM437503	8157	61.15
21654_T	7674944	7674945	G	A	SNV	S	rs397516435	COSM99665	4486	49.35
21665_T	7675215	7675219	CTTG	C	indel	ID	rs879254214	COSM4735425	22643	45.34
21683_T	7674928	7674929	A	T	SNV	S	NR	COSM3742472	36087	33.42

© 2020 Paracchini L et al. *JAMA Network Open.*

eAppendix 3. Droplet Digital Polymerase Chain Reaction Experiments

ddPCR was performed using a QX100TM Droplet Digital PCR system (Bio-Rad) according to the manufacturer's instructions. *TP53* mutated assays, automatically designed on the Bio-Rad online tool, are available upon request. For each assay, the probe for the detection of the target mutated allele was marked with FAM, while the probe for the wild-type allele was marked with HEX. Up to 20 ng of DNA from tumor, blood and Pap Test samples was added to a mixture containing 10 µl of Supermix for Probes (Bio-Rad) and 1 µl of the primer and probe mixture, reaching the final reaction volume of 20 µl. Droplets were generated using the Bio-Rad Automated-Droplet Generator where the Droplet Generation Oil for Probes (Bio-Rad) was added to each reaction mix and the final mixture was transferred in a 96-well plate. Droplets underwent PCR using the following conditions: 5 minutes at 95°C, 40 cycles of 94°C for 30s, 55°C for 1 minute followed by 98°C for 10 minutes (Ramp Rate 2°C/sec). The final PCR product of the generated droplets was analyzed with the QX200 Droplet Reader (Bio-Rad) for fluorescent measurements of FAM and HEX probes. The ddPCR data were analyzed with QuantaSoft analysis Pro software (Bio-Rad) to obtain the Fractional Abundance (FA) of the mutated alleles in the wild-type background. The number of positive and negative droplets is used to calculate the amount of the mutated and reference DNA sequences and their Poisson-based 95% confidence intervals, as previously shown[7].

For each *TP53* mutated assay, corresponding tumor and blood DNA were included in each experiment as positive and negative controls, respectively. They were used to set the thresholds to define positive and negative droplets in each assay. Since the abundance of tumor DNA in Pap test samples was expected to be very low, it was necessary to set is the minimum percentage of tumor content detectable for each assay, in order to estimate its limit of detection. To perform this, the tumor DNA harboring the specific *TP53* variant detected by each assay was serially diluted with a pool of wild-type blood sample from 100% to 0,01%, following these intermediate percentages: 50%, 25%, 10%, 5%, 1%, 0.5%, 0.1% and 0.05%. Given the different DNA integrity between FFPE material and blood, DNA from blood was enzymatically digested using the KAPA fragmentation kit (Roche) for ten minutes at 37°C and then purified with 3X Ampure XP beads (Beckman Coulter). Equally fragmented and concentrated FFPE and blood DNA was then diluted following the previously mentioned scheme. Each tumor DNA dilution was analyzed in triplicate. The lower limit of detection of each assay was defined as the last tumor DNA dilution for which all the replicates contained droplets with only FAM-marked DNA. The relative abundance (RA) was defined as the ratio between the number of FAM-marked droplets and total number of FAM and/or HEX-marked droplets (droplets without any fluorescence signal were not included).

Depending on DNA availability, DNA from Pap Test on healthy women was analyzed twice with all the *TP53* assays to evaluate the presence of these mutations as background artefacts. Each patient's Pap test DNA sample was analyzed twice only with the matched-tumor *TP53* assay. Pap test sample was defined mutated if droplets containing only FAM-marked DNA were detected in both replicates and if the RA was higher than the lowest RA detected by serial dilutions experiments.

© 2020 Paracchini L et al. *JAMA Network Open.*

eTable 2. Serial Dilutions Experiments

To establish the lower limit of detection of each *TP53* assay, dilution experiments were performed as described. For each dilution, the mean value of Relative Abundance (RA) percentage and Standard Deviation (SD) of three independent replicates is reported**.** ND*, not all the replicates presented FAM droplets; ND, Not Detected.

***TP53* mutation**	**Tumor DNA serial dilutions (mean RA % ± SD)**			
	**0.1%**	**0.05%**	**0.01%**	**0.00%**
c.818G>A p.R273H	0.15 ± 0.09	0.07 ± 0.04	ND*	ND*
c.817C>T p.R273C	0.042 ± 0.024	ND*	0.02 ± 0.003	ND*
c.281C>A p.S94*	0.07 ± 0.004	0.05 ± 0.016	ND*	ND
c.469G>T p.V157F	ND	ND*	ND	ND
c.574C>T p.Q192*	0.12 ± 0.04	0.05 ± 0.04	ND	ND
c.820G>T p.V274F	0.25 ± 0.14	0.08 ± 0.07	ND*	ND
c.844C>T p.R282W	0.03 ± 0.01	0.019 ± 0.003	ND*	ND*
c.425_427del p.P142_V143del_insL	0.03 ± 0.02	0.04 ± 0.01	ND	ND
c.993+2T>G	0.04 ± 0.03	0.05 ± 0.03	ND	ND
c.1025G>C p.R342P	ND*	0.04 ± 0.03	ND*	ND
c. 393_395del p.N131del	0.06 ± 0.02	ND*	ND	ND
c. 722 C>G p.S241C	0.02 ± 0.01	ND*	ND*	ND
c.586 C>T p.R196*	0.06 ± 0.04	0.03 ± 0.01	ND*	ND
c.602 T>A p.L201*	0.07 ± 0.02	ND*	ND*	ND

© 2020 Paracchini L et al. *JAMA Network Open.*

eTable 3. Assay Specificity

For each *locus*-specific *TP53* mutation assay generated for ddPCR evaluation, specificity was measured in DNA purified form PAP tests withdrawn from eleven healthy women (N1 to N3 and CTRL1 to CTRL8), as described above. ND*, one of the two replicates showed one FAM-positive droplet, ND, Not Detected, no FAM-positive droplets detected; NA, Not Available due to not sufficient DNA

***TP53* mutation**	**Healthy women' Pap tests**										
	**N1**	**N3**	**N8**	**CTRL 1**	**CTRL 2**	**CTRL 3**	**CTRL 4**	**CTRL 5**	**CTRL 6**	**CTRL 7**	**CTRL 8**
c.818G>A p.R273H	ND	ND	ND	ND	**ND***	ND	ND	ND	ND	**ND***	ND
c.817C>T p.R273C	**ND***	**ND***	ND	**ND***	NA	ND	ND	ND	ND	ND	**ND***
c.281C>A p.S94*	NA	NA	NA	ND	ND	ND	ND	ND	ND	ND	ND
c.469G>T p.V157F	NA	NA	NA	ND	ND	ND	ND	ND	ND	ND	**ND***
c.574C>T p.Q192*	NA	NA	NA	**ND***	ND	ND	ND	ND	ND	ND	ND
c.820G>T p.V274F	ND	ND	ND	ND	ND	ND	ND	ND	ND	ND	ND
c.844C>T p.R282W	ND	**ND***	ND	**ND***	**ND***	ND	ND	**ND***	ND	ND	ND
c.425_427del p.P142_V143del_insL	NA	NA	NA	ND	ND	ND	ND	ND	ND	ND	ND
c.993+2T>G	ND	ND	ND	ND	ND	ND	ND	ND	ND	ND	ND
c.1025G>C p.R342P	NA	NA	NA	ND	ND	ND	ND	ND	ND	ND	ND
c.393_395del p.N131del	ND	ND	ND	ND	ND	ND	ND	ND	ND	ND	ND
c. 722 C>G p.S241C	NA	NA	NA	ND	ND	ND	ND	ND	ND	ND	ND
c.586 C>T p.R196*	NA	NA	NA	ND	ND	ND	ND	ND	NA	**ND***	**ND***
c.602 T>A p.L201*	ND	ND	ND	ND	ND	ND	ND	ND	ND	ND	ND

© 2020 Paracchini L et al. *JAMA Network Open.*

eReferences

1. Li H, Durbin R. Fast and accurate long-read alignment with Burrows-Wheeler transform. *Bioinformatics*. 2010; 26(5):589–595.
2. Cibulskis K, Lawrence M, Carter S et al. Sensitive detection of somatic point mutations in impure and heterogeneous cancer samples. *Nat Biotechnol* 2013; 31: 213–219.
3. Lai Z, Markovets A, Ahdesmaki M, et al. VarDict: a novel and versatile variant caller for next-generation sequencing in cancer research. *Nucleic Acids Res*. 2016; 44(11): e108.
4. McLaren W, Gil L, Hunt SE, et al. The Ensembl Variant Effect Predictor. *Genome Biol* 2016; 17(1): 122.
5. Paila U, Chapman BA, Kirchner R, Quinlan AR. GEMINI: integrative exploration of genetic variation and genome annotations. *PLoS Comput Biol* 2013; 9(7): e1003153.
6. Bouaoun L, Sonkin D, Ardin M, et al. TP53 Variations in Human Cancers: New Lessons from the IARC TP53 Database and Genomics Data. *Hum Mutat* 2016 Sep;37(9):865-76
7. Hindson BJ, Ness KD, Masquelier DA et al. High-throughput droplet digital PCR system for absolute quantitation of DNA copy number. *Anal Chem* 2011; 83: 8604-8610.

© 2020 Paracchini L et al. *JAMA Network Open.*
